# Metabolomic data on molecular weight fractions of the cultivated fruiting body of *Ophiocordyceps sinensis* and their pharmacological effects on airway tissues

**DOI:** 10.1016/j.dib.2025.111904

**Published:** 2025-07-22

**Authors:** Han-Ni Booi, Neng-Yao Goh, Mei-Kee Lee, Chyan-Leong Ng, Szu-Ting Ng, Chon-Seng Tan, Kuan-Hon Lim, Richard Roberts, Shin-Yee Fung, Kang-Nee Ting

**Affiliations:** a School of Pharmacy, University of Nottingham Malaysia, Semenyih, Selangor, Malaysia; b Medicinal Mushroom Research Group, Department of Molecular Medicine, Faculty of Medicine, Universiti Malaya, Kuala Lumpur, Malaysia; c Institute of Systems Biology, Universiti Kebangsaan Malaysia, Bangi, Selangor, Malaysia; d LiGNO Biotech Sdn. Bhd., Balakong Jaya, Selangor, Malaysia; e School of Life Sciences, University of Nottingham Medical School, Queen’s Medical Centre, Nottingham, United Kingdom

**Keywords:** Medicinal mushroom, Airway relaxation, Organ bath, Fractionation, Liquid chromatography-mass spectrometry

## Abstract

*Ophiocordyceps sinensis* is a medicinal mushroom that has been traditionally used for promoting respiratory health and addressing diverse therapeutic needs.

The data in this article provides insights into the metabolic profile of the cultivated fruiting body of *Ophiocordyceps sinensis* (xOs™) across different molecular weight (MW) fractions. The cold-water extract obtained from xOs™ was fractionated by MW into high, medium and low MW fractions using size-exclusion chromatography. The metabolic profile of each fraction was analysed through liquid chromatography-mass spectrometry. The identification of metabolites in xOs™ was validated by analysing the mass spectra of both precursor (MS) and daughter ions (MS2) with those documented in the mass spectrometry reference libraries. Additionally, the role of each metabolite was hypothesized based on existing literature to provide scientific rationale for the traditional applications of xOs™.

Further investigations into the fractions of xOs™ were conducted using the organ bath approach, in which the airway relaxant effects of each fraction on isolated airway tissues from adult male Sprague-Dawley rats were examined. This information could shed light on the bioactivity of different xOs™ fractions in relaxing airway smooth muscles. Taken together, the metabolomics and pharmacological dataset of xOs™ presented in this study may serve as a reference to facilitate comparative metabolomic analyses involving *Ophiocordyceps sinensis* and also to support targeted isolation of bioactive components in future works.

Specifications Table

**Table:** 

Subject	Health Sciences, Medical Sciences & Pharmacology
Specific subject area	Ethnopharmacology
Type of data	Graph, figures and tables of analysed LC-ESI-TOF-MS Data
Data collection	**For LC-MS data** Raw data obtained from MicroTOF QIII Bruker Daltonics (Bruker Corp., USA) using an ESI positive ionization coupled with UltiMate 3000 UHPLC system (Dionex) (Thermo Fisher Scientific Corp., USA). **For organ bath experiments** Force transducer (MLTF050/ST, ADInstruments Corp., USA) to detect differences in isometric tension; PowerLab data acquisition programme (LabChart v8.1.16, ADInstruments) for data recording; Prism version 10.4.1 (Graphpad software, USA) for data analysis.
Data source location	Sample of cultivated fruiting body of *Ophiocordyceps sinensis* obtained from LiGNO Biotech Sdn. Bhd., Malaysia. The experiments were conducted at both the University of Nottingham Malaysia and Universiti Kebangsaan Malaysia.
Data accessibility	The data is presented in this article. The dataset is published under the following title on Mendeley Data: Supplementary Data: Metabolomic Data on Molecular Weight Fractions of the Cultivated Fruiting Body of *Ophiocordyceps sinensis* and Their Pharmacological Effects on Airway Tissues Preview of the supplementary data is available at: https://data.mendeley.com/datasets/9jbkdjj7nr/1
Related research article	–

## 1 Value of the Data

• This study provides a foundational LC-MS dataset showcasing the metabolic profile of the cultivated fruiting body of *Ophiocordyceps sinensis* (xOs™) across different molecular weight fractions, providing additional support for its longstanding use in traditional applications.
• This dataset offers insights into the putatively identified metabolites and their relative abundance across xOs™ fractions, serving as an important reference for future isolation work.
• This dataset reveals the pharmacological effects of different molecular weight fractions of xOs™ on airway tissues obtained from adult male Sprague-Dawley rats, highlighting the bioactivity of each fraction on airway responsiveness.
• With preliminary insights into the metabolic profile of each fraction, this dataset is valuable for substantiating mechanistic studies and provides a basis for future studies to elucidate the full mechanisms of action of xOs™ across its diverse therapeutic applications.

## 2 Background

*Ophiocordyceps sinensis* is an entomopathogenic fungus renowned in traditional Chinese medicine, particularly for its role in promoting respiratory health [[1], [2], [3]]. Due to overexploitation and habitat loss, it has been listed as one of the endangered species on the China Biodiversity Red List 2018 [4]. Therefore, cultivated variants have been introduced as a sustainable alternative to meet the growing market need [5]. While significant advancement has been achieved, the bioactive components of the cultivated variants remain largely unexplored. In this study, a cultivated strain of the *Ophiocordyceps sinensis* (xOs™) was used as a safe and sustainable alternative. The nutrient and chemical analysis of the xOs™ were previously published in [6]. xOs™ was then subjected to cold-water extraction to further standardize the bioactive constituents present in the samples [7]. To date, there is no available data on the metabolomics and pharmacological profiles of the different fractions of xOs™. Therefore, this study highlights the findings of fractioning the xOs™ into three different MW fractions: high (> 13.7 kDa), medium (2.2–12.5 kDa) and low (< 1.8 kDa). The organ bath technique was used to assess the airway relaxant properties [8], while liquid chromatography-mass spectrometry (LC-MS) was performed to obtain an extensive metabolic profile for each fraction [9]. These findings facilitate the identification of bioactive components and their correlation with the pharmacological properties of each fraction, which may lead to potential isolation work in the future.

## 3 Data Description

**Fig. 1:**
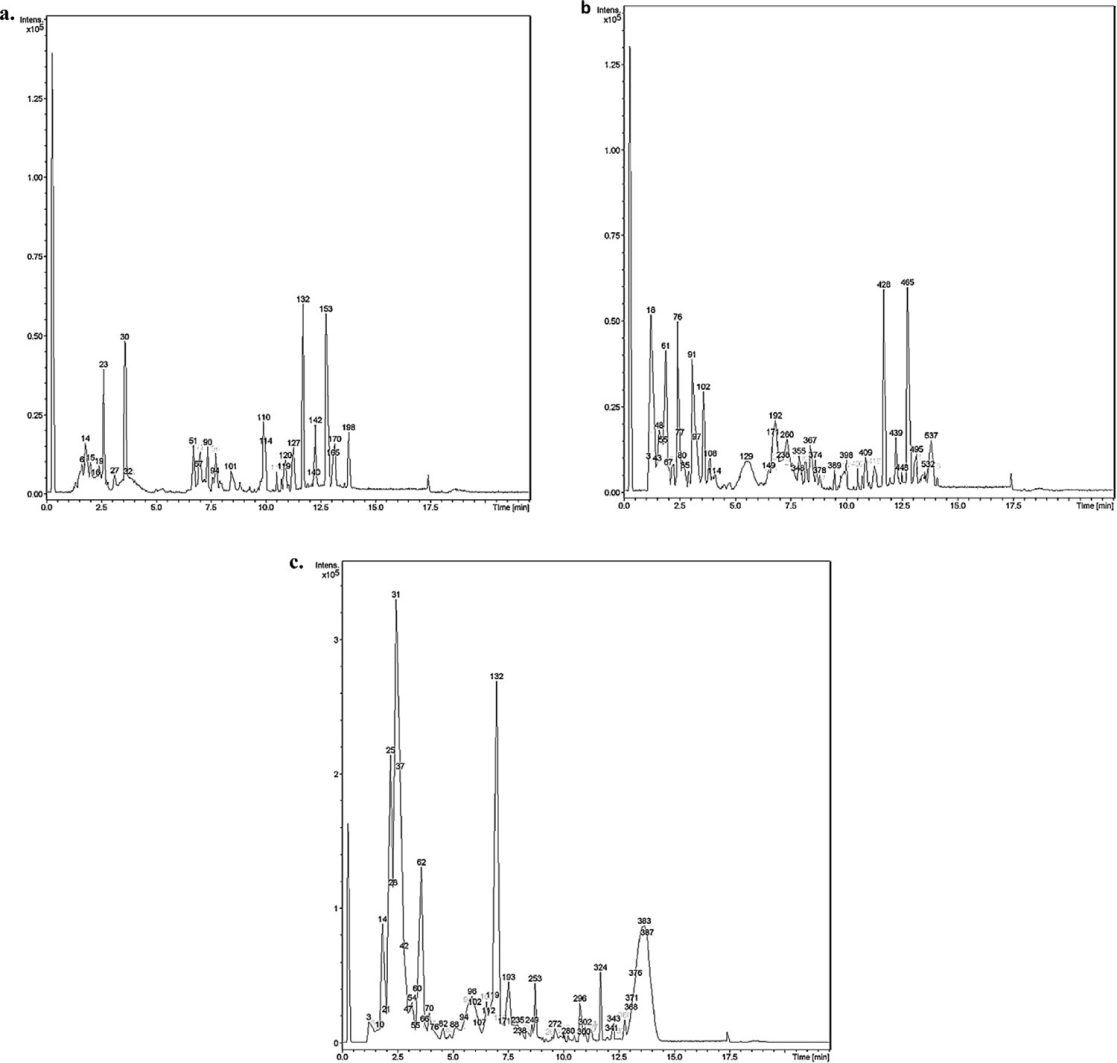
BPC obtained from LC-MS analysis for xOs™: (a) HMW fraction, (b) MMW fraction and (c) LMW fraction in positive ionization mode. The chromatograms were acquired using a MicroTOF QIII Bruker Daltonics mass spectrometer over a run time of 22 min. Prominent peaks were numbered in ascending order based on their retention time (in minutes) on the x-axis, while the y-axis represented compound intensity in units of x 10^5^. Distinct peak profiles were observed for each xOs™ fraction [(a), (b) and (c)], as indicated by variations in peak distribution and intensity. Fig. 1: The BPC for (a) HMW fraction displays multiple sharp and well-resolved peaks spanning the entire retention time range, with a notable increase in compositional density in the late-eluting region beyond 10.0 min.; (b) MMW fraction shows prominent peaks in both the early (≤5.0 min) and late (≥10.0 min) retention time ranges, with a number of broad peaks in the mid-retention time range (5.1–9.9 min).; (c) LMW demonstrates multiple high-intensity peaks in the early retention time range, followed by numerous smaller peaks and a prominent broad peak in the late retention time range.

The LC-MS data for different fractions of xOs™ are represented as base peak chromatogram (BPC) in positive ionization mode, as illustrated in Figs. 1aFig. 1, Fig. 1 for HMW, MMW and LMW, respectively.

The LC-MS analysis provides putative identification of metabolites for each fraction, as presented in Supplementary Data 1: Table S1a, S1b and S1c, corresponding to the respective high, medium and low MW fractions. Table S1 in Supplementary Data 1 includes the following parameters: peak number (denoted by ‘a'), retention time (provided in minutes), metabolite identification, molecular formula, theoretical mass (given in mass to charge ratio), parent ion (the intact compound) [M^+^H]^+^, mass error (expressed in parts per million), fragment formula, fragment ion (ionized fragment), identification level (denoted by ‘b'), relative abundance (intensity), reference, along with suggested functions for each metabolite. In this study, a tentative identification of 38 metabolites was documented for HMW, 50 metabolites were identified in MMW and 98 metabolites were characterized in LMW.

**Fig. 2:**
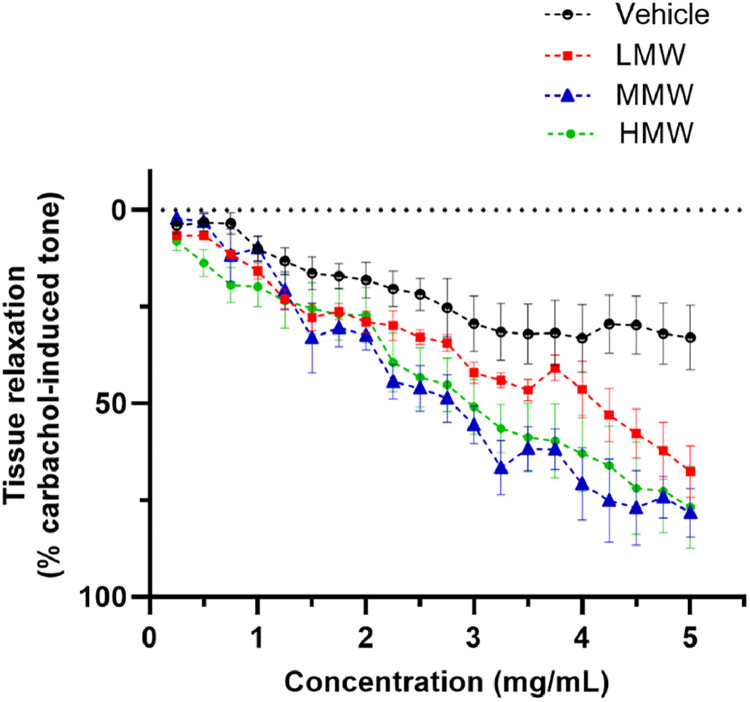
Effect of different MW fractions of xOs™ (HMW, MMW and LMW) on pre-contracted isolated rat tracheal rings. The magnitude of airway relaxation induced by the xOs™ fractions was observed in the following order: MMW > HMW > LMW. Tissue responses were represented as a percentage of carbachol-induced contraction and were expressed as mean ± SEM from 5 animals.

Apart from identifying the metabolites present in the fractions of xOs™, Fig. 2 illustrates the bioactivity of each fraction in relaxing airway smooth muscles, as assessed using the organ bath technique, presented in graphical form. The E_max_ values for each fraction are detailed in Table 1.

**Table 1:** The maximum tissue relaxation response (E_max_) obtained for each xOs™ fraction, expressed as means ± SEM of 5 animals. An unpaired *t-*test was performed between the vehicle and each xOs™ fraction group (^⁎⁎^*p* < 0.01, ^⁎⁎⁎^*p* < 0.001).

Treatment group	E_max_ ( %)
Vehicle	31.60 ± 7.84
HMW	69.14 ± 10.76^⁎⁎⁎^
MMW	71.39 ± 7.18^⁎⁎⁎^
LMW	57.57 ± 8.10^⁎⁎^

## 4 Experimental Design, Materials and Methods

### 4.1 Preparation of xOs™ cold-water extract

Cultivated fruiting body powder of xOs™ was provided by LiGNO Biotech Sdn. Bhd. (Selangor, Malaysia). The cold-water extract of xOs™ was prepared as described in [10].

### 4.2 Fractionation of xOs™ cold-water extract

The fractionation approach was adapted from the methods outlined in [11]. 1 g of xOs™ cold-water extract was dissolved in 10 ml of 50 mM ammonium acetate. The resulting mixture was loaded into the conventional column of [40 cm × (1.25 cm)2 × π], packed with Sephadex G50 fine (Sigma-Aldrich, USA). 50 mM of ammonium acetate was used as the buffer to elute the samples loaded by gravity. 100 fractions of 2.5 ml each were collected.

The eluted fractions were pooled into 3 major fractions based on their MW, namely high MW (HMW; > 13.7 kDa), medium MW (MMW; 2.0–12.5 kDa), and low MW (LMW; < 1.8 kDa). The MW of the pooled fractions was determined by comparing the ratio of elution volume (Ve) and void volume (Vo) to Ve/Vo of the selected protein standards including bovine serum albumin (66 kDa), egg albumin (45 kDa), soybean trypsin inhibitor (20.1 kDa) and apotinin (6.5 kDa). The pooled fractions were then treated with lyophilization and store at −20 °C for subsequent studies.

### 4.3 Liquid chromatography-mass spectrometry

The chromatographic separation of each xOs™ fraciton (HMW, MMW and LMW) was conducted in accordance with the methodology detailed in [10]. The separation ultilised a Thermo Scientific C18 column (AcclaimTM Polar Advantage II, 3 × 150 mm, 3 µm particle size) connected to an UltiMate 3000 UHPLC system (Dionex) (Thermo Fisher Scientific Corp., USA). Gradient elution was conducted using water with 0.1 % formic acid as solvent A and 100 % acetonitrile as solvent B, at a flow rate of 0.4 mL/min and a column temperature of 40 °C, with a total run time of 22 min. Each sample was injected at a volume of 1 µL. The elution gradient began at 5 % of solvent B for the first 3 min, followed by an increment to 80 % of solvent B from 3 to 10 min. The mobile phase composition was maintained at 80 % of solvent B from 10 to 15 min followed by a reduction to 5 % between 15 and 22 min to facilitate column re-equilibration.

Samples were analysed using the MicroTOF QIII Bruker Daltonics (Bruker Corp., USA), equipped with an electrospray ionization (ESI) source operating in positive ion mode, calibrated for high-resolution mass acquisition under the following conditions: capillary voltage, 4500 V; nebulizer pressure, 1.2 bar; drying gas, 8 L/min at 200 °C. The mass range was set at 50–1000 m*/z*. Accurate mass data of the molecular ions were obtained from the TOF analyser and were subsequently processed using Compass Data Analysis software (Bruker Daltonics). Identification of compound for each fraction was performed through spectral matching of both precursor (MS) and daughter ions (MS2) to those reference spectra in MassBank, KEGG, HMDB, NIH, PubChem, ChemSpider and MoNA libraries, with a mass error threshold of <20 ppm.

## 5 Organ bath experiments

### 5.1 Animals

The experiments were performed using male Sprague-Dawley rats, selected within a weight range of 250 – 400 g and aged between 3 and 4 months. Animals were sourced from the Animal House, Universiti Kebangsaan Malaysia and euthanized on the experimental day through carbon dioxide asphyxiation.

### 5.2 Tissue preparation

The organ bath experiment was set up as described in [8], employing 2 mm tracheal rings isolated from the male Sprague-Dawley rats.

### 5.3 Organ bath experimental protocol

The tracheal rings were pre-contracted with 1 µM of carbachol to induce a stable submaximal contractile tone. Cumulative concentration-response curves to different fractions of xOs™ (HMW, MMW, and LMW) were constructed at 15-minute intervals or until the tissue response reached a plateau. Purified water was served as the vehicle control throughout the experiment. The magnitude of tissue relaxation was quantified as a fraction of the contraction induced by 1 µM of carbachol and expressed as a percentage of the carbachol-induced tone.

### 5.4 Statistical analysis

Each xOs™ fraction was expressed in mg/mL. Data was expressed as the mean ± standard error of mean (SEM), based on the number of animals used (*n*). Nonlinear regression analysis was applied to each cumulative concentration-response curve to determine the maximum contraction or relaxation response (E_max_). In cases where a plateau was not observed and E_max_ could not be determined, the response at the highest concentration tested was taken as the E_max_ value. Statistical analysis was performed using an unpaired *t*-test, with *p*-values <0.05 considered statistically significant. All data were analysed using Prism version 10.4.1 (Graphpad software, USA).

## Limitations

The use of untargeted LC-MS metabolomics in this study enables broad detection of metabolites across xOs™ fractions, providing preliminary insights into the metabolic profile of the xOs™ in each fraction. The detected metabolites across xOs™ fractions were putatively identified as structural elucidation of each individual compound requires additional techniques such as nuclear magnetic resonance (NMR), which are resource-intensive and not readily scalable for high-throughput applications such as those conducted in this study. To complement these findings, the nutrient and chemical analysis of the xOs™ fruiting body can be found in [6].

## Ethics Statement

Ethics approval for this study was granted by the University of Nottingham's Animal Welfare and Ethics Review Body (AWERB), under approvals UNMC26 and UNMC310. All procedures were conducted in full compliance with the relevant European directives governing the use of animals in scientific research (Directive 86/609/EEC).

## Credit Author Statement

**Han Ni Booi:** Conceptualization, Data curation, Formal analysis, Investigation, Writing – original draft. **Neng Yao Goh:** Data curation, Writing – review & editing. **Mei Kee Lee, Kuan Hon Lim, Richard Roberts:** Supervision, Resources. **Szu Ting Ng, Chon Seng Tan:** Funding acquisition. **Shin Yee Fung, Chyan Leong Ng, Kang Nee Ting:** Conceptualization, Methodology, Supervision, Project administration, Resources, Writing – review & editing.

## Data Availability

Mendeley DataSupplementary Data: Metabolomic Data on Molecular Weight Fractions of the Cultivated Fruiting Body of Ophiocordyceps sinensis and Their Pharmacological Effects on Airway Tissues (Original data).

## Appendix Supplementary materials

Image, application 1
