# Supplementary material for: Metabolomic data on molecular weight fractions of the cultivated fruiting body of Ophiocordyceps sinensis and their pharmacological effects on airway tissues
Source: Data Brief. 2025 Jul 22;62:111904. doi: 10.1016/j.dib.2025.111904 (PMC12332881; doi:10.1016/j.dib.2025.111904)
Supplement: Supplementary file 1 [file mmc1.docx]

**Supplementary Data 1**

**Table S1: Putative identification of metabolites in xOs^TM^ at different fractions: (a) HMW (b) MMW and (c) LMW.** The reference standard for the mass spectra was acquired from MassBank, KEGG, HMDB, NIH, PubChem, ChemSpider and MoNA libraries. ^a^Peak number, correspond to the elution order in the BPC and ^b^Level of identification (ID level) is classified as follows: 1- identification verified using authentic standard; 2- identification done at fragmentation level (MS/MS) by matching with online libraries; 3- identification done using parent ion (m/z value) only due to absence of fragments. The mass chromatograms for each compound are provided in Supplementary Data 2. (Abbreviations: ID, level of identification; RT, retention time; MS, mass-spectra).

**Table S1(a):**

| Peak no.^a^ | RT (min) | Compound/Metabolite ID | Molecular formula | Theoretical mass (m/z) | Parent ion (m/z) [M+H]^+^  /[M+H-H_2_O]^+^ | Mass error (ppm) | Fragment formula | Fragment ion [M+H]^+^ | ID level^b^ | Relative abundance (intensity) | Reference | Function |
| --- | --- | --- | --- | --- | --- | --- | --- | --- | --- | --- | --- | --- |
| 2 | 1.3 | Lys-Thr | C_10_H_21_N_3_O_4_ | 248.160483 | 248.1602 | -1.1 | - | - | 3 | 1561 | MassBank | Dipeptide |
| 3 | 1.3 | Lys-Ser | C_9_H_19_N_3_O_4_ | 234.144833 | 234.143 | -7.8 | - | - | 3 | 1449 | MassBank | Dipeptide |
| 5 | 1.4 | 6-(alpha-D-Glucosaminyl)-1D-myo-inositol | C_12_H_23_NO_10_ | 342.139472 | 342.1396 | 0.4 | - | - | 3 | 711 | KEGG | Secondary metabolites that may serve a role as defence or signalling molecules |
| 6 | 1.6 | L-Arginine | C_6_H_14_N_4_O_2_ | 175.118952 | 175.1184 | -3.2 | [C_5_H_10_NO_2_]^+^  [C_6_H_12_N_3_O_2_]^+^ | 116.0709  158.0929 | 2 | 6715 | MassBank | Precursor for nitric oxide formation |
| 10 | 1.8 | Galactose | C_6_H_12_O_6_ | 198.097210 | 198.0961 | -5.6 | [C_8_H_9_N-4H]^+^  [C_9_H_11_NO-4H]^+^  [C_9_H_11_NO-2H]^+^  [C_13_H_15_N-5H]^+^ | 115.0399  145.0491  163.0605  180.0862 | 2 | 16132 | MoNA | Incorporated into N- and O-linked glycans |
| 11 | 1.8 | Ala-Pro | C_8_H_14_N_2_O_3_ | 187.107719 | 187.1066 | -6 | [C_5_H_8_NO_2_+H]+H^+^ | 116.0704 | 2 | 7585 | HMDB | Dipeptide |
| 12 | 1.8 | L-Glutamic acid | C_5_H_9_NO_4_ | 148.060434 | 148.0592 | -8.3 | [C_5_H_9_NO_3_-H]^+^  [C_5_H_9_NO_4_+H]^+^ | 130.0541  148.0511 | 2 | 7919 | HMDB | Precursor of GABA, the main inhibitory neurotransmitter |
| 13 | 1.8 | Gln-Pro | C_10_H_17_N_3_O_4_ | 244.129183 | 244.1287 | -2 | [C_5_H_8_NO_2_+H]+H^+^[C_9_H_14_N_2_O_2_-H]^+^  [C_10_H_14_N_2_O_3_-H]^+^  [C_10_H_16_N_3_O_3_]^+^  [C_10_H_15_N_2_O_4_]^+^ | 116.0709  181.0973  209.0906  226.1185  227.1044 | 2 | 4851 | HMDB | Dipeptide |
| 14 | 1.8 | Methionine | C_5_H_11_NO_3_S | 166.053240 | 166.0535 | 1.6 | - | - | 3 | 6405 | HMDB | Antioxidant |
| 17 | 2.0 | Val-Pro | C_10_H_18_N_2_O_3_ | 215.139019 | 215.138 | -4.7 | [C_5_H_8_NO_2_+H]+H^+^  [C_9_H_17_N_2_O]^+^ | 116.0705  169.1349 | 2 | 10117 | HMDB | Dipeptide |
| 19 | 2.3 | L-leucine | C_6_H_13_NO_2_ | 132.101905 | 132.1018 | -0.8 | - | - | 3 | 6709 | HMDB | Muscle growth and regulate blood glucose level |
| 22 | 2.6 | Tyrosine | C_9_H_11_NO_3_ | 182.08117 | 182.0807 | -2.6 | [C_8_H_8_O-H]^+^ [C_7_H_7_O_2_]^+^  [C_8_H_10_NO]^+^  [C_9_H_8_O_2_-H]^+^  [C_9_H_9_O_3_]^+^ | 119.0489  123.0446  136.0761  147.0434  165.0550 | 2 | 39624 | HMDB | Synthesis of hormones and neurotransmitters |
| 25 | 2.9 | Tyr-Pro | C_14_H_18_N_2_O_4_ | 279.133934 | 279.1332 | -2.7 | - | - | 3 | 1351 | HMDB | Dipeptide |
| 26 | 3.1 | Glu-Ile | C_11_H_20_N_2_O_5_ | 261.144498 | 261.1437 | -3.1 | - | - | 3 | 1752 | HMDB | Dipeptide |
| 27 | 3.1 | Leu-Pro | C_11_H_20_N_2_O_3_ | 229.154669 | 229.1544 | -2.1 | [C_5_H_8_NO_2_+H]+H^+^ | 116.0704 | 2 | 4717 | MassBank | Dipeptide |
| 29 | 3.4 | Tyr-Val | C_14_H_20_N_2_O_4_ | 281.149584 | 281.1482 | -4.9 | - | - | 3 | 1348 | HMDB | Dipeptide |
| 30 | 3.6 | Phenylalanine | C_9_H_11_NO_2_ | 166.086255 | 166.0856 | -3.9 | [C_8_H_8_-H]^+^  [C_8_H_10_N-H]^+^  [C_8_H_10_N]^+^  [C_9_H_8_O-H]^+^  [C_9_H_9_O_2_]^+^ | 103.0544  119.0725  120.0813  131.0481  149.0589 | 2 | 47927 | MassBank | Production of neurotransmitters: norepinephrine and dopamine |
| 40 | 5.0 | Trp-Asp | C_15_H_17_N_3_O_5_ | 320.124097 | 320.1235 | -1.9 | - | - | 3 | 1669 | HMDB | Dipeptide |
| 50 | 6.7 | Tryptophan | C_11_H_12_N_2_O_2_ | 205.097154 | 205.0968 | -1.7 | [C_8_H_6_N+H]+H^+^  [C_9_H_8_N+H]+H^+^  [C_10_H_9_N]+H^+^  [C_10_H_11_N_2_]^+^ [C_11_H_9_NO-H]^+^  [C_11_H_10_NO_2_]^+^ | 118.0641  132.0796  144.0802  159.1062  170.0600  188.0701 | 2 | 18061 | HMDB | Growth and precursor of serotonin |
| 67 | 6.9 | Glu-Ala-Gly-Ile-Gln | C_21_H_36_N_6_O_9_ | 517.261653 | 517.2589 | -5.3 | - | - | 3 | 3725 | NIH | Oligopeptide |
| 89 | 7.3 | Val-Trp | C_16_H_21_N_3_O_3_ | 304.165568 | 304.1632 | -7.8 | [C_7_H_11_NO_3_+H]+H^+^[C_11_H_10_NO_2_]^+^ | 159.0908  188.0691 | 2 | 14937 | HMDB | Dipeptide |
| 94 | 7.7 | 2-[(Z)-[(4Z)-4-(Diaminomethylidenehydrazinylidene)hexan-3-ylidene]amino]guanidine | C_8_H_18_N_8_ | 227.172719 | 227.1727 | -0.1 | [C_5_H_9_N_5_]^+^ | 139.0851 | 2 | 4549 | HMDB | - |
| 95 | 7.7 | 1,2,3,4-Tetrahydro-beta-carboline-3-carboxylic acid | C_12_H_12_N_2_O_2_ | 217.097154 | 217.0965 | -3 | - | - | 3 | 1796 | HMDB | Antioxidant |
| 98 | 8.0 | N-Methyl-1H-indole-3-propanamide | C_12_H_14_N_2_O | 203.117890 | 203.1177 | -0.9 | - | - | 3 | 1119 | HMDB | Tryptamine derivatives |
| 108 | 9.7 | Fumigaclavine B | C_16_H_20_N_2_O | 257.164840 | 257.1646 | -0.9 | - | - | 3 | 2108 | HMDB | Antibacterial |
| 111 | 9.9 | Phytosphingosine | C_18_H_39_NO_3_ | 318.300271 | 318.2965 | -11.8 | [C_17_H_37_NO-H]^+^  [C_18_H_37_NO-H]^+^  [C_18_H_39_NO_3_+H]^+^ | 270.2748  282.2745  318.2972 | 2 | 22873 | MoNA | Structural component of cell membrane |
| 126 | 11.3 | N-(2,2-dimethylthian-4-yl)-2-morpholin-4-yl-N-phenylacetamide | C_15_H_28_N_2_O_7_ | 349.194425 | 349.1945 | 0.2 | - | - | 3 | 11246 | NIH | - |
| 127 | 11.3 | (2R,3R,4R,5S)-6-[3-[bis(2-hydroxyethyl)amino]propylamino]hexane-1,2,3,4,5-pentol | C_13_H_30_N_2_O_7_ | 327.212578 | 327.2121 | -1.5 | [C_7_H_16_N_2_O_2_-H]^+^  [C_9_H_20_N_2_O_4_-H]^+^  [C_9_H_20_N_2_O_5_]+H^+^  [C_13_H_28_N_2_O_5_-H]^+^ | 159.1143  219.1332  237.1425  291.1907 | 2 | 6430 | PubChem | - |
| 142 | 12.2 | 1-(2-Methylprop-2-enyl)-2-[(2-methyltriazol-4-yl)methyl]guanidine | C_9_H_16_N_6_ | 209.150921 | 209.1507 | -1.1 | [C_7_H_10_N_3_-H]^+^  [C_5_H_9_N_6_]^+^  [C_8_H_13_N_6_]^+^ | 135.0787  153.0897  193.1179 | 2 | 18645 | PubChem | - |
| 148 | 12.7 | (9Z,12E)-15,16-dihydroxyoctadeca-9,12-dienoic acid | C_18_H_32_O_4_ | 313.237336 | 313.2327 | -8.9 | [C_9_H_12_+H]^+^  [C_9_H_16_O-H]^+^  [C_10_H_17_O_3_]^+^  [C_18_H_32_O_4_+H]^+^ | 121.0989  139.1100  185.1159  313.2301 | 2 | 23163 | MoNA | Oxylipin, derivative of linoleic acid |
| 150 | 12.7 | N-[(6-tert-butyl-2-cyclohexylsulfanylpyridin-3-yl)methyl]propanamide | C_19_H_30_N_2_OS | 335.215161 | 335.2151 | -0.2 | [C_13_H_19_N_2_OS]^+^ | 251.1230 | 2 | 56933 | PubChem | - |
| 152 | 12.7 | (3-hydroxyhexadecanoyl)glycine | C_18_H_35_NO_4_ | 330.263885 | 330.2584 | -17.6 | [C_9_H_14_O+H]^+^  [C_10_H_12_O+H]^+^ | 139.1084  149.1007 | 2 | 9489 | MoNA | Minor metabolite in fatty acid metabolism |
| 153 | 12.7 | 13-Oxooctadecadienoic acid | C_18_H_30_O_3_ | 295.220000 | 295.2215 | 5.1 | [C_9_H_16_O+H]^+^  [C1_8_H_32_O_3_-H]^+^ | 141.1262  295.2190 | 2 | 9249 | MoNA | Bioactive oxylipin derived from linoleic acid |
| 156 | 12.9 | LysoPE(0:0/20:2(11Z,14Z)) | C_25_H_48_NO_7_P | 506.324116 | 506.3250 | 1.7 | - | - | 3 | 1365 | HMDB | Building blocks for membrane biogenesis |
| 165 | 13.1 | Soyasaponin I | C_48_H_78_O_18_ | 943.526092 | 943.5124 | -14.5 | [C_6_H_10_O_3_-H]^+^  [C_15_H_20_O+H]^+^ | 129.0522  217.1887 | 2 | 8644 | COCONUT | Anticancer |
| 170 | 13.1 | 2-[[1,3-dihydroxy-2-(hydroxymethyl)propan-2-yl]amino]-N-(2-methylpropyl)acetamide | C_10_H_22_N_2_O_4_ | 235.165234 | 235.1651 | -0.6 | [C_6_H_13_N_2_O_4_+H]+H^+^ | 179.1044 | 2 | 15980 | PubChem | - |
| 174 | 13.3 | Ganolucidic acid C | C_30_H_46_O_7_ | 519.331630 | 519.3304 | -2.4 | - | - | 3 | 1018 | HMDB | Anti-inflammatory, anticancer, anti-diabetic, and anti-obesity |
| 199 | 13.8 | Ala-Gln-Asn | C_12_H_21_N_5_O_6_ | 332.156460 | 332.1532 | -9.81 | - | - | 3 | 1327 | PubChem | Oligopeptide |

**Table S1(b):**

| Peak no.^a^ | RT (min) | Compound/Metabolite ID | Molecular formula | Theoretical mass (m/z) | Parent ion (m/z) [M+H]^+^ | Mass error (ppm) | Fragment formula | Fragment ion [M+H]^+^ | ID level^b^ | Relative abundance (intensity) | Reference | Function | |
| --- | --- | --- | --- | --- | --- | --- | --- | --- | --- | --- | --- | --- | --- |
| 8 | 1.2 | 2,6-Di(dodecan-2-yl)-4-nonylphenol | C_39_H_72_O | 557.565593 | 557.5669 | 2.34 | - | - | 3 | 7739 | NIH | | - |
| 21 | 1.2 | [(2R)-2-[(2-methylpropan-2-yl)oxycarbonylamino]propyl] 4-[(2-oxo-3,4-dihydro-1H-quinolin-6-yl)oxy]butanoate | C_21_H_30_N_2_O_6_ | 407.217663 | 407.2168 | -2.1 | [C_7_H_9_O]+H^+^  [C_8_H_16_NO_3_]+H^+^  [C_13_H_14_NO_2_]+H^+^  [C_16_H_19_O_4_+H]+H^+^  [C_16_H_25_NO_4_-H]^+^ | 110.0716  175.1197  217.1197  277.1417  294.6608 | 2 | 35995 | NIH | | - |
| 27 | 1.2 | Octyl 2,3,4,6-O-Tetraacetyl-beta-D-mannopyranoside | C_22_H_36_O_10_ | 461.238124 | 461.2392 | 2.34 | - | - | 3 | 1268 | NIH | | - |
| 28 | 1.2 | Protactin | C_32_H_48_N_6_O_8_ | 645.360639 | 645.3632 | 3.97 | - | - | 3 | 10267 | NIH | | Oligopeptide |
| 45 | 1.6 | N6,N6,N6-Trimethyl-L-lysine | C_9_H_20_N_2_O_2_ | 189.159754 | 189.1608 | 5.5 | [C_6_H_12_NO_2_]^+^  [C_9_H_20_N_2_O-H]^+^ | 130.0865  171.1480 | 2 | 4694 | HMDB | | Precursor of carnitine and a coenzyme of fatty acid oxidation |
| 48 | 1.6 | H-Arg-Val-Arg-al | C_17_H_35_N_9_O_3_ | 414.293563 | 414.2938 | 0.6 | [C_6_H_13_N_4_O]^+^  [C_10_H_20_N_4_O-H]^+^  [C_16_H_31_N_6_O_2_-2H]^+^  [C_16_H_31_N_7_O_2_]+H^+^  [C_17_H_33_N_8_O_2_-2H]^+^  [C_17_H_35_N_9_O_2_-H]^+^ | 157.1090  211.1552  337.2365  354.2628  379.2577  396.2830 | 2 | 7581 | PubChem | | Oligopeptide |
| 56 | 1.8 | Glycerophosphocholine | C_8_H_20_NO_6_P | 258.110100 | 258.1112 | 4.3 | [C_5_H_13_NO]+H^+^  [C_2_H_4_O_4_P+H]+H^+^  [C_5_H_13_NO_4_P+H]+H^+^ | 104.1071  125.0010  184.0746 | 2 | 6193 | HMDB | | Neurotransmission (acetylcholine), transmembrane signalling, synthesis of phospholipids in and fat and cholesterol metabolism |
| 61 | 1.9 | Choline sulfate | C_5_H_13_NO_4_S | 184.063805 | 184.0642 | 2.1 | [C_5_H_13_NO]+H^+^ | 104.1073 | 2 | 41386 | PubChem | | Regulate memory, mood and muscle control |
| 62 | 1.9 | beta-D-galacto-hexopyranosyl-(1->3)-[2-acetamido-2-deoxy-beta-D-gluco-hexopyranosyl-(1->6)]-beta-D-galacto-hexopyranosyl-(1->4)-beta-D-gluco-hexopyranose | C_26_H_45_NO_21_ | 708.255684 | 708.2589 | 4.5 | [C_6_H_9_NO_2_-H]^+^  [C_6_H_11_O_5_]^+^  [C_8_H_13_NO_4_-H]^+^  [C_8_H_14_NO_5_]^+^  [C_8_H_14_NO_6_+H]+H^+^ | 126.0555  163.0603  186.0778  204.0864  222.0966 | 2 | 7766 | HMDB | | Energy source |
| 63 | 1.9 | 4-[(5-hydrazinylthiadiazol-4-yl)methyl]-N-propylmorpholine-3-carboxamide | C_11_H_20_N_6_O_2_S | 301.144121 | 301.1440 | -0.4 | [C_5_H_9_N_3_-H]^+^  [C_6_H_10_N_5_S]^+^  [C_7_H_10_N_4_O_2_+2H]+H^+^  [C_7_H_12_N_5_S-H]^+^  [C_11_H_20_N_5_O_2_]+H^+^ | 110.0709  184.0645  185.1025  197.0722  255.1700 | 2 | 7001 | PubChem | | - |
| 64 | 1.9 | Valine | C_5_H_11_NO_2_ | 118.086255 | 118.0870 | 6.31 | - | - | 3 | 2631 | NIH | | Stimulate muscle growth and regeneration and involved in energy production |
| 67 | 2.0 | Ala-Leu | C_9_H_18_N_2_O_3_ | 203.139019 | 203.139 | -0.09 | [C_6_H_9_NO_3_]+H^+^  [C_7_H_12_NO_3_]^+^ | 144.0662  158.0809 | 2 | 6969 | HMDB | | Dipeptide |
| 70 | 2.1 | Val-Val | C_10_H_20_N_2_O_3_ | 217.154669 | 217.1551 | 1.98 | [C_9_H_19_N_2_O]^+^ | 171.1494 | 2 | 5558 | HMDB | | Dipeptide |
| 75 | 2.5 | Gly-ala-prolinamide | C_10_H_18_N_4_O_3_ | 243.145167 | 243.1472 | 2.8 | [C_7_H_12_O_2_]^+^  [C_9_H_14_NO_3_]^+^ | 128.0837  184.0982 | 2 | 49857 | ChemSpider | | Oligopeptide |
| 77 | 2.5 | Pyrosaccharopine | C_11_H_18_N_2_O_5_ | 259.128848 | 259.1307 | 7.1 | [C_7_H_13_N_2_O+H]+H^+^  [C_9_H_13_NO_2_]+H^+^ [C_10_H_16_N_2_O_2_-H]^+^  [C_10_H_16_N_2_O_2_]+H^+^ [C_10_H_17_N_2_O_3_]^+^ | 143.1168  168.1037  195.1129  197.1302  213.1246 | 2 | 13760 | HMDB | | Proline derivative and involve in amino acid metabolism |
| 78 | 2.6 | N-(1-Deoxy-I(2)-D-fructopyranos-1-yl)-L-isoleucine | C_12_H_23_NO_7_ | 294.154729 | 294.1562 | 5 | [C_6_H_12_NO_2_+H]+H^+^  [C_7_H_14_NO_2_]^+^  [C_11_H_21_NO_4_-H]^+^  [C_11_H_22_NO_5_]^+^  [C_12_H_21_NO_5_-H]^+^  [C_12_H_22_NO_6_]+H^+^ | 132.1018  144.1010  230.1401  248.1503  258.1350  277.1510 | 2 | 4626 | PubChem | | Isoleucine derivative |
| 80 | 2.6 | Tyrosine | C_9_H_11_NO_3_ | 182.08117 | 182.0819 | 4 | [C_8_H_8_O-H]^+^  [C_7_H_7_O_2_]^+^  [C_8_H_10_NO]^+^  [C_9_H_8_O_2_-H]^+^  [C_9_H_9_O_3_]^+^ | 119.0486  123.0448  136.0767  147.0447  165.0557 | 2 | 7894 | PubChem | | Production of neurotransmitters: Dopamine and norepinephrine, improve cognitive function |
| 83 | 2.7 | Hydroxyprolyl-Lysine | C_11_H_21_N_3_O_4_ | 260.160483 | 260.1617 | 4.7 | [C_6_H_12_NO_2_+H]+H^+^ [C_10_H_19_N_3_O-H]^+^  [C_10_H_18_N_2_O_2_-H]^+^ [C_10_H_16_N_2_O_3_]+H^+^ | 132.1022  196.1436  197.1278  213.1250 | 2 | 6705 | HMDB | | Dipeptide |
| 84 | 2.7 | Val-Ala | C_8_H_16_N_2_O_3_ | 189.123369 | 189.1231 | -1.42 | - | - | 3 | 1599 | NIH | | Dipeptide |
| 85 | 2.7 | Thr-Leu | C_10_H_20_N_2_O_4_ | 233.149584 | 233.1509 | 5.6 | [C_5_H_7_NO_3_]+H^+^  [C_6_H_12_NO_2_+H]+H^+^ [C_9_H_19_N_2_O_2_]^+^ | 130.0495  132.1016  187.1453 | 2 | 4705 | HMDB | | Dipeptide |
| 90 | 3.1 | Glu-Leu | C_11_H_20_N_2_O_5_ | 261.144498 | 261.146 | 5.8 | [C_6_H_12_NO_2_+H]+H^+^ [C_11_H_19_N_2_O_4_]^+^ | 132.1017  243.1365 | 2 | 5146 | HMDB | | Dipeptide |
| 91 | 3.2 | Citric acid | C_6_H_8_O_7_ | 193.034279 | 193.0348 | 2.7 | [C_5_H_6_O_4_-H]^+^  [C_6_H_6_O_5_-H]^+^ | 129.0189  157.0126 | 2 | 28632 | HMDB | | Antioxidant |
| 92 | 3.1 | Amino beta-D-gluco-hexopyranosiduronic acid | C_6_H_11_NO_7_ | 210.060828 | 210.0614 | 2.7 | [C_5_H_7_O_4_-2H]^+^  [C_5_H_8_O_5_-H]^+^  [C_6_H_8_O_6_-H]^+^ | 129.0189  147.0290  175.0242 | 2 | 29012 | PubChem | | Sugar acid derivative |
| 95 | 3.2 | 2,5-Dimethyl-3-(methyldithio)furan | C_7_H_10_OS_2_ | 175.024583 | 175.0246 | 0.1 | - | - | 3 | 4225 | HMDB | | - |
| 96 | 3.2 | Aconitic acid | C_6_H_6_O_6_ | 175.023714 | 175.0246 | 5.06 | - | - | 3 | 4225 | NIH | | Involve in cellular energy production |
| 97 | 3.3 | Leu-Val | C_11_H_22_N_2_O_3_ | 231.170319 | 231.1703 | -0.1 | [C_5_H_10_NO_2_]^+^ [C_5_H_10_NO_2_+H]+H^+^  [C_10_H_21_N_2_O]^+^ | 116.0689  118.0854  185.1668 | 2 | 11926 | HMDB | | Dipeptide |
| 102 | 3.6 | Phenylalanine | C_9_H_11_NO_2_ | 166.086255 | 166.0867 | 2.7 | [C_8_H_8_-H]^+^  [C_8_H_10_N]^+^  [C_9_H_8_O-H]^+^  [C_9_H_9_O_2_]^+^ | 103.0547  120.0814  131.0496  149.0591 | 2 | 23559 | HMDB | | Production of neurotransmitters: norepinephrine and dopamine |
| 104 | 3.7 | Leu-Pro | C_11_H_20_N_2_O_3_ | 229.154669 | 229.1544 | -1.17 | - | - | 3 | 2667 | HMDB | | Dipeptide |
| 118 | 4.3 | Phe-His-Leu | C_21_H_29_N_5_O_4_ | 416.229231 | 416.2296 | 0.89 | - | - | 3 | 1795 | ChemSpider | | Oligopeptide |
| 128 | 5.5 | 2-[2-Acetamidoethyl-[2-(2,3-dihydroindol-1-yl)-2-oxoethyl]amino]propanoic acid | C_17_H_23_N_3_O_4_ | 334.176133 | 334.1772 | 3.2 | [C_8_H_8_N+H]+H^+^  [C_6_H_12_N_2_O]+H^+^  [C_7_H_14_N_2_O-H]^+^  [C_6_H_12_N_2_O_2_+2H]+H^+^ [C_7_H_13_N_2_O_3_]^+^ | 120.0800  129.1022  141.1022  147.1113  173.0912 | 2 | 2369 | HMDB | | - |
| 150 | 6.5 | Arg-Gln-Trp | C_22_H_32_N_8_O_5_ | 489.256843 | 489.2585 | 3.39 | - | - | 3 | 1839 | HMDB | | Oligopeptide |
| 153 | 6.6 | Glu-Pro-Lys | C_16_H_28_N_4_O_6_ | 373.208161 | 373.2095 | 3.59 | - | - | 3 | 1882 | NIH | | Oligopeptide |
| 159 | 6.6 | Glycyl-L-isoleucyl-N-[di(1H-imidazol-2-yl)methyl]glycinamide | C_17_H_26_N_8_O_3_ | 391.220063 | 391.2212 | 2.91 | - | - | 3 | 2162 | NIH | | Oligopeptide |
| 165 | 6.7 | [2-amino-5-[(2-amino-3,4-dioxo-cyclobuten-1-yl)amino]pentyl] N-[5-amino-1-(carbamoyloxymethyl)pentyl]carbamate | C_17_H_30_N_6_O_6_ | 415.229959 | 415.2287 | -3 | [C_6_H_12_N_2_O]+H^+^  [C_6_H_12_N_2_O_2_+2H]+H^+^  [C_12_H_25_N_3_O_3_]+H^+^ | 129.1011  147.1112  260.1955 | 2 | 4911 | PubChem | | - |
| 180 | 6.7 | Leu-Cys-Val-Pro | C_19_H_34_N_4_O_5_S | 431.232267 | 431.2325 | 0.5 | - | - | 3 | 2638 | PubChem | | Oligopeptide |
| 183 | 6.8 | 14alpha-Hydroxypaxilline | C_27_H_33_NO_5_ | 452.243150 | 452.2425 | -1.4 | - | - | 3 | 2638 | HMDB | | - |
| 186 | 6.8 | Val-Asp-Ser-Lys | C_18_H_33_N_5_O_8_ | 448.240190 | 448.2412 | 2.25 | - | - | 3 | 2760 | NIH | | Oligopeptide |
| 198 | 6.8 | Gln-His-Ile | C_17_H_28_N_6_O_5_ | 397.219395 | 397.2186 | -2 | - | - | 3 | 3988 | NIH | | Oligopeptide |
| 206 | 6.9 | H-Phe-Ala-Pro-Gly-Trp-NH_2_ | C_30_H_37_N_7_O_5_ | 576.292894 | 576.2913 | -2.77 | - | - | 3 | 2634 | NIH | | Oligopeptide |
| 209 | 6.9 | Lys-Trp-Trp | C_28_H_34_N_6_O_4_ | 519.271430 | 519.2713 | -0.25 | - | - | 3 | 3249 | NIH | | Oligopeptide |
| 210 | 6.9 | Ser-Gly-Ala-Gly-Lys-Thr | C_20_H_37_N_7_O_9_ | 520.272552 | 520.2724 | -0.29 | - | - | 3 | 3924 | NIH | | Oligopeptide |
| 211 | 7.0 | 2-[[(5S)-5-amino-6-oxohexyl]-(diaminomethylideneamino)amino]guanidine | C_8_H_20_N_8_O | 245.183284 | 245.1821 | -4.8 | [C_2_H_8_N_7_+H]+H^+^ | 132.1003 | 2 | 9028 | PubChem | | - |
| 213 | 7.0 | (2S,4R,5R,6R)-6-[(1R)-2-[[(2S)-2-[[(2S)-2-aminopropanoyl]amino]-5-(diaminomethylideneamino)pentanoyl]amino]-1-hydroxyethyl]-4,5-dihydroxyoxane-2-carboxylic acid | C_17_H_32_N_6_O_8_ | 449.235438 | 449.2342 | -2.8 | [C_5_H_9_O_3_+2H]+H^+^  [C_6_H_12_N_2_O]+H^+^  [C_5_H_9_O_4_+2H]+H^+^  [C_7_H_14_N_2_O-H]^+^  [C_7_H_13_N_5_O_2_+H]+H^+^ | 120.0783  129.1008  136.0740  141.1024  201.1202 | 2 | 3823 | PubChem | | Oligopeptide  (H-Ala-Arg-Unk) |
| 227 | 7.1 | (2S)-2-[[(2S)-2-[[(2R)-6-amino-2-[[2-(4-phenyldiazenylphenyl)acetyl]amino]hexanoyl]amino]-3-phenylpropanoyl]amino]propanoic acid | C_32_H_38_N_6_O_5_ | 587.297645 | 587.2961 | -2.6 | [C_8_H_9_N]+H^+^  [C_6_H_12_N_2_O]+H^+^  [C_7_H_13_N_3_O_2_+H]+H^+^  [C_11_H_14_N_2_O+H]+H^+^ | 120.0807  129.1016  173.1142  192.1261 | 2 | 6326 | PubChem | | Oligopeptide  (Unk-Lys-Phe-Ala-OH) |
| 230 | 7.1 | Lys-Val-Tyr-Tyr | C_29_H_41_N_5_O_7_ | 572.307875 | 572.3094 | 2.7 | - | - | 3 | 11859 | PubChem | | Oligopeptide |
| 371 | 8.6 | 2-[(8R,11S,14S,17S,20S,24R,27S)-20-(3-amino-3-oxo-propyl)-4,4,8,14,17-pentamethyl-2,3,7,10,13,16,19,22,26-nonaoxo-24-(2-phenylethyl)-6-oxa-1,9,12,15,18,21,25-heptazabicyclo[25.4.0]hentriacontan-11-yl]acetic acid | C_41_H_58_N_8_O_13_ | 871.419610 | 871.4222 | 3 | [C_19_H_29_N_2_O_5_]^+^  [C_23_H_30_N_3_O_4_-H]^+^  [C_24_H_30_N_3_O_5_-H]^+^  [C_19_H_30_N_5_O_7_]^+^  [C_24_H_37_N_6_O_8_]+H^+^  [C_27_H_42_N_7_O_9_-H]^+^  [C_30_H_43_N_8_O_7_+H]+H^+^ | 365.2099  411.2139  439.2098  440.2112  538.2747  607.2948  629.3368 | 2 | 7995 | PubChem | | - |
| 405 | 10.6 | 1-Octen-3-ol-3-o-beta-D-xylopyranosyl(1->6)-beta-D-glucopyranoside | C_19_H_34_O_10_ | 423.222474 | 423.2218 | -1.6 | - | - | 3 | 1846 | KEGG | | Storage and transport of bioactive components |
| 436 | 12.1 | Lys-Trp | C_17_H_24_N_4_O_3_ | 333.192117 | 333.1929 | 2.3 | - | - | 3 | 2273 | HMDB | | Dipeptide |
| 466 | 12.7 | Dihydroxyoctadecadienoic acid | C_18_H_32_O_4_ | 313.228490 | 313.2282 | -0.9 | [C_9_H_18_O-H]^+^  [C_18_H_30_O_2_-H]^+^ | 141.1215  277.2084 | 2 | 26216 | MoNA | | Bioactive oxylipins, derived from linoleic acid |
| 468 | 12.7 | 13-Oxooctadecadienoic acid | C_18_H_30_O_3_ | 295.220000 | 295.2175 | -8.5 | [C_9_H_16_O+H]^+^  [C1_8_H_32_O_3_-H]^+^ | 141.1210  295.2174 | 2 | 10247 | MoNA | | Bioactive oxylipin derived from linoleic acid |

**Table S1(c):**

| Peak no.^a^ | RT (min) | Compound/Metabolite ID | Molecular formula | Theoretical mass (m/z) | Parent ion (m/z) [M+H]^+^ | Mass error (ppm) | Fragment formula | Fragment ion [M+H]^+^ | ID level^b^ | Relative abundance (intensity) | Reference | Function |
| --- | --- | --- | --- | --- | --- | --- | --- | --- | --- | --- | --- | --- |
| 2 | 1.2 | Iminoglycine | C_2_HN_5_O_2_ | 128.020301 | 128.0198 | -3.91 | - | - | 3 | 5927 | NIH | Glycine derivative |
| 3 | 1.2 | (2-Amino-4-methylpentyl) 4-(dihydroxyamino)oxybutanoate | C_10_H_22_N_2_O_5_ | 251.160148 | 251.1591 | -4.17 | [C_10_H_20_N_2_O_3_-H]^+^  [C_10_H_19_NO_4_-H]^+^  [C_10_H_21_N_2_O_4_]^+^  [C_10_H_20_NO_5_]^+^ | 215.2375  216.1221  233.1483  234.1345 | 2 | 10915 | NIH | - |
| 6 | 1.4 | Spermidine | C_7_H_19_N_3_ | 146.165174 | 146.1646 | -3.93 | [C_7_H_15_N-H]^+^ [C_7_H_17_N_2_]^+^ | 112.1116  129.1382 | 2 | 8102 | HMDB/MoNA | Antiaging, antitumour, anti-inflammation, cardioprotective and neuroprotective |
| 8 | 1.5 | L-Histidine trimethylbetaine | C_9_H_15_N_3_O_2_ | 198.123703 | 198.1225 | -6.07 | [C_6_H_6_N_2_O_2_]+H^+^  [C_8_H_15_N_3_]+H^+^ | 139.0497  154.1337 | 2 | 5883 | HMDB | Antioxidant |
| 9 | 1.7 | Histidine | C_6_H_9_N_3_O_2_ | 156.076753 | 156.0757 | -6.75 | - | - | 3 | 1944 | HMDB | Promotes growth and tissue repair |
| 11 | 1.7 | Methyl 2-(2-methyl-1-oxo-pyridin-1-ium-4-ylidene)-3H-oxazole-4-carboxylate | C_11_H_11_N_2_O_4_ | 236.079158 | 236.0794 | 1 | [C_7_H_4_NO_3_-H]^+^  [C_8_H_5_N_2_O_2_]+H^+^ [C_9_H_8_N_2_O_2_]^+^  [C_9_H_8_N_2_O_2_]+H^+^  [C_11_H_10_O_3_+H]+H^+^ | 149.0115  162.0430  176.0584  177.0672  192.0801 | 2 | 6506 | PubChem | - |
| 12 | 1.8 | Valine | C_5_H_11_NO_2_ | 118.086255 | 118.0863 | 0.38 | - | - | 3 | 6293 | HMDB | Promotes muscle growth and tissue repair |
| 15 | 1.8 | Cordycepic acid | C_6_H_14_O_6_ | 183.086315 | 183.0873 | 5.4 | [C_4_H_9_O_3_]^+^  [C_6_H_11_O_4_]^+^ | 104.1062  147.0659 | 2 | 88319 | PubChem | Anti-inflammation |
| 18 | 1.8 | L-arginine | C_6_H_14_N_4_O_2_ | 175.118952 | 175.1183 | -3.7 | [C_5_H_9_N_2_O]^+^H^+^  [C_5_H_11_NO_2_+H]^+^  [C_6_H_12_N_3_O_2_]^+^ | 114.0522  118.0846  128.0717 | 2 | 26390 | MoNA | Nitric oxide production |
| 20 | 2.0 | (2S)-2-(2,6-dioxo-1-piperidyl)-5-guanidino-pentanoic acid | C_11_H_18_N_4_O_4_ | 271.140082 | 271.1392 | -3.3 | [C_9_H_13_NO_2_-H]^+^  [C_9_H_11_NO_3_+2H]+H^+^  [C_10_H_13_N_2_O_3_+H]+H^+^  [C_10_H_14_NO_4_]^+^  [C_11_H_15_N_3_O_3_-H]^+^  [C_11_H_17_N_4_O_3_]^+^ | 166.0851  184.0954  211.1070  212.0930  236.1020  253.1283 | 2 | 21960 | PubChem | L-arginine derivative |
| 21 | 2.0 | Amidinoproline | C_6_H_11_N_3_O_2_ | 158.092403 | 158.0924 | -0.02 | [C_5_H_10_N_3_]^+^ | 112.0856 | 2 | 16452 | NIH | Proline derivative |
| 22 | 2.0 | N-(1-Deoxy-1-fructosyl)valine | C_6_H_21_NO_7_ | 280.139078 | 280.1395 | 1.51 | [C_10_H_19_NO_4_-H]^+^  [C_11_H_20_NO_6_]^+^  [C_11_H_20_NO_6_]+H^+^ | 216.1232  262.1280  263.1347 | 2 | 12377 | HMDB | Valine derivative |
| 24 | 2.0 | Ala-Leu | C_9_H_18_N_2_O_3_ | 203.139019 | 203.1382 | -4 | [C_8_H_15_NO-H]^+^  [C_6_H_9_NO_3_-H]^+^ | 140.1073  142.0492 | 2 | 16548 | PubChem | Dipeptide |
| 25 | 2.2 | Gly-Gly-Lys-Val | C_15_H_29_N_5_O_5_ | 360.224146 | 360.2237 | -1.24 | [C_5_H_10_NO_2_+H]+H^+^  [C_9_H_15_NO_3_-H]^+^  [C_10_H_19_N_4_O_3_]^+^  [C_11_H_18_N_2_O_4_+2H]+H^+^ | 118.0867  184.0969  243.1446  245.1477 | 2 | 17589 | PubChem | Oligopeptide |
| 26 | 2.2 | Glycyl-L-alanyl-L-prolinamide | C_10_H_18_N_4_O_3_ | 243.145167 | 243.1450 | -0.69 | C_5_H_8_N_2_O_2_  C_5_H_9_N_2_O_2_  C_8_H_14_N_3_O_2_  C_8_H_15_N_3_O_2_ | 128.0823  129.0838  184.0972  185.0997 | 2 | 303280 | ChemSpider | Oligopeptide |
| 30 | 2.4 | Isoleucine | C_6_H_13_NO_2_ | 132.101905 | 132.1022 | 2.23 | - | - | 3 | 41445 | HMDB | Improves glucose metabolism and immune function |
| 31 | 2.4 | 5-acetamido-3,5-dideoxy-4-O-methyl-D-glycero-D-galacto-non-2-ulosonic acid | C_12_H_21_NO_9_ | 324.128908 | 324.1283 | -1.88 | [C_6_H_11_NO_2_+2H]+H^+^  [C_9_H_16_O_6_-H]^+^  [C_9_H_18_NO_5_+2H]+H^+^ | 132.1007  219.0850  223.1405 | 2 | 13216 | HMDB, NIH | Sialic acid derivative |
| 34 | 2.6 | Amino beta-D-gluco-hexopyranosiduronic acid | C_6_H_11_NO_7_ | 210.060828 | 210.0607 | -0.6 | [C_5_H_7_O_4_-2H]^+^  [C_5_H_8_O_4_-2H]^+^  [C_5_H_8_O_5_-H]^+^  [C_6_H_8_O_6_-H]^+^ | 129.0182  130.0257  147.0283  175.0228 | 2 | 84555 | PubChem | Detoxification |
| 35 | 2.6 | Tyrosine | C_9_H_11_NO_3_ | 182.08117 | 182.0813 | 0.71 | [C_8_H_8_O-H]^+^  [C_7_H_7_O_2_]^+^  [C_8_H_10_NO]^+^  [C_9_H_8_O_2_-H]^+^  [C_9_H_9_O_3_]^+^ | 119.0489  123.0444  136.0758  147.0438  165.0545 | 2 | 202165 | HMDB | Precursor of thyroid hormones, and melanin |
| 36 | 2.6 | N-(1-Deoxy-beta-D-fructopyranos-1-yl)-L-isoleucine | C_12_H_23_NO_7_ | 294.154729 | 294.1532 | -5.2 | [C_6_H_12_NO_2_+H]+H^+^ [C_7_H_14_NO_2_]^+^  [C_6_H_11_NO_4_]^+^  [C_11_H_21_NO_4_-H]^+^  [C_11_H_22_NO_5_]^+^  [C_12_H_21_NO_5_-H]^+^  [C_12_H_22_NO_6_]^+^ | 132.1016  144.1007  161.0690  230.1380248.1482  258.1319  276.1434 | 2 | 32597 | HMDB | Glycosylated amino acid |
| 38 | 2.6 | (4-aminobenzoyl)-DL-glutamic acid | C_12_H_15_N_2_O_5_ | 268.105373 | 268.1041 | -4.7 | [C_7_H_8_N_2_O]^+^  [C_9_H_11_N_2_O+H]+H^+^  [C_11_H_14_N_2_O_2_+2H]+H^+^ | 136.0618  165.1018  209.1278 | 2 | 159235 | MassBank | Involved in learning and memory |
| 44 | 2.8 | N6-Acetyl-L-lysine | C_8_H_16_N_2_O_3_ | 189.123369 | 189.1224 | -5.12 | [C_5_H_8_NO_3_]^+^  [C_6_H_9_NO_3_-H]^+^ | 130.0495  142.0495 | 2 | 20022 | HMDB | Protein synthesis |
| 45 | 2.8 | 5-Methyl-uridine | C_10_H_14_N_2_O_6_ | 259.092463 | 259.0910 | -5.6 | [C_5_H_11_NO_3_-3H]^+^  [C_9_H_13_NO_3_+H]^+^ | 130.0502  184.0961 | 2 | 41623 | MoNA | Modified nucleoside |
| 50 | 3.2 | Glu-Leu | C_11_H_20_N_2_O_5_ | 261.144498 | 261.1438 | -2.67 | [C_6_H_12_NO_2_+H]+H^+^  [C_10_H_18_N_2_O_2_-H]^+^  [C_11_H_18_N_2_O_3_-H]^+^  [C_11_H_19_N_2_O_4_]^+^ | 132.1022  197.1276  225.1228  243.1323 | 2 | 20400 | HMDB | Dipeptide |
| 52 | 3.2 | Citric acid | C_6_H_8_O_7_ | 193.034279 | 193.0336 | -3.5 | [C_5_H_6_O_4_-H]^+^  [C_6_H_8_O_4_-5H]^+^  [C_6_H_8_O_5_-3H]^+^ | 129.0165  139.0034  157.0094 | 2 | 8970 | HMDB | Antioxidant |
| 54 | 3.2 | Leu-Pro | C_11_H_20_N_2_O_3_ | 229.154669 | 229.1526 | -9 | [C_5_H_9_NO_2_+H]^+^  [C_5_H_10_NO_2_+H]^+^ | 116.0708  117.0774 | 2 | 5668 | PubChem | Dipeptide |
| 57 | 3.4 | Guanosine | C_10_H_13_N_5_O_5_ | 284.098945 | 284.0981 | -2.97 | [C_4_H_2_N_3_O+H]+H^+^  [C_5_H_9_O_4_]^+^  [C_5_H_2_N_4_O]+H^+^  [C_5_H_4_N_5_+H]+H^+^  [C_5_H_4_N_5_O+H]+H^+^  [C_6_H_7_N_3_O_2_]^+^ | 110.0337  133.0495  135.0305  136.0626  152.0569  153.0544 | 2 | 37296 | MassBank | Neuroprotective |
| 58 | 3.4 | Guanine | C_5_H_5_N_5_O | 152.056686 | 152.0525 | -1.22 | [C_4_H_3_N_3_O]+H^+^ | 110.0362 | 2 | 7606 | HMDB | Building block |
| 59 | 3.9 | Val-Leu | C_11_H_22_N_2_O_3_ | 231.170319 | 231.1692 | -4.84 | [C_5_H_9_NO_2_]+H^+^  [C_5_H_9_NO_2_+H]+H^+^  [C_5_H_9_NO_2_+2H]+H^+^  [C_6_H_12_NO_2_+H]+H^+^  [C_10_H_21_N_2_O]^+^ | 116.0709  117.0790  118.0863  132.1018  185.1639 | 2 | 26007 | HMDB | Dipeptide |
| 61 | 3.6 | Indoline | C_8_H_9_N | 120.080776 | 120.0811 | 2.7 | - | - | 3 | 8104 | HMDB | - |
| 62 | 3.6 | Phenylalanine | C_9_H_11_NO_2_ | 166.086255 | 166.0864 | 0.87 | [C_8_H_8_-H]^+^  [C_8_H_10_N-H]^+^  [C_8_H_10_N]^+^  [C_9_H_8_O-H]^+^  [C_9_H_9_O_2_]^+^ | 103.0536  119.0735  120.0814  131.0485  149.0584 | 2 | 130790 | HMDB | Production of neurotransmitters: norepinephrine and dopamine |
| 63 | 3.6 | Tyr-Val | C_14_H_20_N_2_O_4_ | 281.149584 | 281.1487 | -3.14 | [C_5_H_10_NO_2_]^+^  [C_8_H_9_N]+H^+^  [C_8_H_10_NO]^+^ | 116.0720  120.0794  136.0749 | 2 | 4420 | HMDB | Dipeptide |
| 71 | 3.9 | Gly-Phe | C_11_H_14_N_2_O_3_ | 223.107719 | 223.1071 | -2.77 | [C_8_H_9_N]+H^+^  [C_9_H_9_O_2_]^+^  [C_9_H_10_NO_2_+H]+H^+^ [C_10_H_13_N_2_O]^+^ | 120.0810  149.0587  166.0854  177.1015 | 2 | 7815 | HMDB | Dipeptide |
| 74 | 4.0 | 5-Deoxymyricanone | C_21_H_24_O_4_ | 341.174736 | 341.1746 | -0.4 | - | - | 3 | 2630 | HMDB | - |
| 75 | 4.0 | Ala-Phe | C_12_H_16_N_2_O_3_ | 237.123369 | 237.1226 | -3.24 | [C_8_H_9_N]+H^+^  [C_9_H_10_NO_2_+H]+H^+^ | 120.0807  166.0851 | 2 | 11121 | HMDB | Dipeptide |
| 76 | 4.2 | N-(1-Deoxy-1-fructosyl)phenylalanine | C_15_H_21_NO_7_ | 328.139078 | 328.1396 | 1.59 | [C_8_H_9_N]+H^+^  [C_9_H_11_N-H]^+^  [C_9_H_10_NO_2_+H]+H^+^  [C_10_H_12_NO_2_]^+^ [C_14_H_19_NO_4_-H]^+^  [C_15_H_19_NO_5_-H]^+^  [C_15_H_20_NO_6_]^+^ | 120.0807  132.0809  166.0854  178.0862  264.1221  292.1171  310.1277 | 2 | 6621 | HMDB | - |
| 77 | 4.2 | Thr-Phe | C_13_H_18_N_2_O_4_ | 267.133934 | 267.1329 | -3.87 | [C_8_H_10_N]^+^  [C_9_H_11_N_2_O]^+^  [C_12_H_16_N_2_O-H]^+^  [C_12_H_17_N_2_O_2_]^+^  [C_13_H_17_N_2_O_3_]^+^ | 120.0820  163.0881  203.1174  221.1300  249.1226 | 2 | 6104 | HMDB | Dipeptide |
| 79 | 4.4 | Pyridoxamine | C_8_H_12_N_2_O_2_ | 169.097154 | 169.0959 | -7.42 | - | - | 3 | 2065 | HMDB | Vitamin B6 derivative |
| 84 | 4.8 | Pyroglutamylproline | C_10_H_14_N_2_O_4_ | 227.102633 | 227.1021 | -2.35 | - | - | 3 | 4658 | HMDB | Dipeptide |
| 85 | 4.9 | Glu-Phe | C_14_H_18_N_2_O_5_ | 295.128848 | 295.1289 | 0.18 | [C_8_H_9_N]+H^+^  [C_9_H_10_NO_2_+H]+H^+^ [C_13_H_16_N_2_O_2_-H]^+^  [C_14_H_17_N_2_O_4_]^+^ | 120.0813  166.0862  231.1111  277.1173 | 2 | 4425 | HMDB | Dipeptide |
| 87 | 5.0 | Pantothenic acid | C_9_H_17_NO_5_ | 220.117949 | 220.1168 | -5.22 | [C_4_H_6_NO_3_]^+^  [C_9_H_15_NO_3_-H]^+^  [C_9_H_16_NO_4_]^+^ | 116.0357  184.0959  202.1069 | 2 | 8930 | HMDB | Vitamin B5 |
| 89 | 5.1 | Phe-Val | C_14_H_20_N_2_O_3_ | 265.154669 | 265.1532 | -5.5 | [C_8_H_11_N-H]^+^  [C_11_H_13_N_2_O_2_+H]+H^+^  [C_13_H_20_N_2_O-H]^+^ | 120.0810  207.1113  219.1479 | 2 | 8297 | HMDB | Dipeptide |
| 90 | 5.3 | Leu-Tyr | C_15_H_22_N_2_O_4_ | 295.165234 | 295.1648 | -1.47 | C_8_H_8_O  C_8_H_10_NO  C_9_H_12_N_2_O_3_ | 120.0821  136.0720  182.0779 | 2 | 5595 | HMDB | Dipeptide |
| 93 | 5.5 | Isopropylmaleic acid | C_7_H_10_O_4_ | 159.065185 | 159.0659 | 4.5 | [C_6_H_9_O_2_]^+^  [C_7_H_9_O_3_]^+^ | 113.0603  141.0554 | 2 | 13540 | HMDB | - |
| 94 | 5.5 | 2-Aminoheptanedioic acid | C_7_H_13_NO_4_ | 176.091734 | 276.0920 | 1.51 | [C_6_H_10_O_2_-H]^+^  [C_7_H_10_O_3_-H]^+^  [C_7_H_11_O_4_]^+^  [C_7_H_11_O_4_]+H^+^ | 113.0602  141.0555  159.0650  160.0719 | 2 | 9853 | HMDB | Defence/ signalling molecule |
| 95 | 5.7 | 2-methoxyethyl (2S)-1-[(2S)-2-[[(2R)-3-cyclopentylsulfanyl-1-ethoxy-1-oxopropan-2-yl]amino]propanoyl]pyrrolidine-2-carboxylate | C_21_H_36_N_2_O_6_S | 445.236684 | 445.2376 | 2.1 | [C_6_H_11_N_2_O+H]+H^+^  [C_6_H_11_NO_2_]+H^+^  [C_7_H_11_N_2_O_3_+H]+H^+^  [C_15_H_24_N_2_O_5_+2H]+H^+^ | 129.1020  130.0869  173.0909  315.1923 | 2 | 19051 | PubChem | Oligopeptide  (Unk-Ala-Pro-OetOMe) |
| 96 | 5.9 | Leu-Val-Cys | C_14_H_27_N_3_O_4_S | 334.179504 | 334.1799 | 1.2 | [C_6_H_13_N_2_O]^+^  [C_6_H_9_NO_2_+2H]+H^+^  [C_7_H_14_N_2_O-H]^+^  [C_7_H_12_N_2_O_3_]+H^+^  [C_8_H_15_N_3_O_2_]+H^+^ | 129.1018  130.0874  141.1006  173.0936  186.1232 | 2 | 24884 | PubChem | Oligopeptide |
| 108 | 6.3 | Glu-Ile-Ser | C_14_H_25_N_3_O_7_ | 348.176527 | 348.1751 | -4.1 | - | - | 3 | 4407 | HMDB | Oligopeptide |
| 119 | 6.8 | Tryptophan | C_11_H_12_N_2_O_2_ | 205.097154 | 205.0968 | -1.73 | [C_8_H_6_N+H]+H^+^  [C_9_H_8_N+H]+H^+^  [C_10_H_9_N^]+^  [C_10_H_9_N]+H^+^  [C_10_H_11_N_2_]^+^  [C_11_H_9_NO-H]^+^  [C_11_H_10_NO_2_]^+^ | 118.0658  132.0808  143.0733  144.0804  159.0916  170.0603  188.0706 | 2 | 22634 | HMDB | Growth |
| 124 | 6.8 | 1-[[(E)-(5-hydroxyindol-3-ylidene)methyl]amino]-2-pentylguanidine | C_15_H_21_N_5_O | 288.181887 | 288.1827 | 2.82 | - | - | 3 | 2851 | NIH | - |
| 128 | 6.9 | 3-Methyl-2-phenyl-N-(1-phenylpropyl)quinoline-4-carboximidate | C_26_H_24_N_2_O | 381.196140 | 381.1976 | 3.83 | - | - | 3 | 3214 | HMDB | - |
| 134 | 7.0 | Ala-pro-ser-ala-ala | C_17_H_29_N_5_O_7_ | 416.213975 | 416.2149 | 3.83 | [C_7_H_10_NO_3_+2H]+H^+^  [C_12_H_20_N_4_O_3_]+H^+^ | 159.0902  269.1596 | 2 | 22318 | NIH/HMDB | Oligopeptide |
| 147 | 7.1 | Phe-Pro | C_14_H_18_N_2_O_3_ | 263.139019 | 263.1393 | 1.07 | [C_5_H_8_NO_2_+H]+H^+^ [C_8_H_10_N]^+^ | 116.0707  120.0808 | 2 | 8450 | HMDB | Dipeptide |
| 155 | 7.1 | Succinyladenosine | C_14_H_17_N_5_O_8_ | 384.114989 | 384.1164 | 3.67 | [C_5_H_3_N_5_+2H]+H^+^  [C_6_H_10_NO_4_+H]+H^+^  [C_7_H_5_N_5_O_2_]+H^+^  [C_8_H_7_N_5_O_2_]+H^+^  [C_9_H_7_N_5_O_3_]+H^+^  [C_9_H_8_N_5_O_4_+H]+H^+^  [C_10_H_10_N_3_O_5_+H]+H^+^ | 136.0626  162.0774  192.0520  206.0664  234.0613  252.0725  254.0770 | 2 | 15477 | HMDB | Aspartic acid derivative |
| 171 | 7.3 | Phe-Tyr | C_18_H_20_N_2_O_4_ | 329.149584 | 329.1494 | -0.56 | [C_8_H_10_N]^+^  [C_8_H_9_NO]+H^+^  [C_13_H_16_N_2_O_2_-H]^+^ | 120.0810  136.0771  231.1129 | 2 | 8707 | HMDB | Dipeptide |
| 178 | 7.3 | H-Tyr(Bn)-N(For)Gly-Gly-Phe-Leu-OH | C_36_H_43_N_5_O_8_ | 674.318440 | 674.3179 | -0.8 | - | - | 3 | 587 | PubChem | Oligopeptide |
| 190 | 7.5 | Ile-Phe | C_15_H_22_N_2_O_3_ | 279.170319 | 279.1691 | -4.37 | [C_8_H_9_N]+H^+^  [C_9_H_10_NO_2_+H]+H^+^  C_9_H_12_NO_2_ | 120.0810  166.0862 | 2 | 38557 | HMDB | Dipeptide |
| 197 | 7.5 | Trp- His-Pro- Gln | C_27_H_34_N_8_O_6_ | 567.267407 | 567.2682 | 1.4 | [C_7_H_10_NO_3_+2H]+H^+^  [C_12_H_18_N_4_O_5_+2H]+H^+^  [C_15_H_20_N_4_O_4_]+H^+^ | 159.0893  301.1503  321.1556 | 2 | 5364 | PubChem | Oligopeptide |
| 204 | 7.6 | (4R,9β,16α)-16-hydroxy-9,10,14-trimethyl-1,11,22-trioxo-20,24-epoxy-4,9-cyclo-9,10-secochola-2,5,23-trien-2-yl β-D-glucopyranoside | C_33_H_44_O_11_ | 617.295639 | 617.2977 | 3.3 | - | - | 3 | 2976 | KEGG | Glycoside |
| 214 | 7.7 | Phe-Ser-Leu | C_18_H_27_N_3_O_5_ | 366.202347 | 366.2015 | -2.31 | [C_8_H_10_N]^+^  [C_8_H_16_N_2_O_2_]+H^+^  [C_11_H_15_N_2_O_2_]^+^  [C_9_H_17_N_2_O_4_+H]+H^+^ | 120.0810  173.1271  207.1128  219.1341 | 2 | 4166 | HMDB | Oligopeptide |
| 215 | 7.7 | Trp-Leu | C_17_H_23_N_3_O_3_ | 318.181218 | 318.1801 | -3.51 | [C_9_H_8_N+H]+H^+^  [C_10_H_9_N]+H^+^  [C_10_H_12_N_2_-H]^+^  [C_11_H_9_NO-H]^+^  [C_17_H_20_N_2_O_3_]+H^+^ | 132.0813  144.0807  159.0915  170.0601  301.1529 | 2 | 4889 | HMDB | Dipeptide |
| 230 | 7.8 | Ala-Ala-Lys-Ala | C_15_H_29_N_5_O_5_ | 360.224146 | 260.2237 | -1.2 | [C_9_H_16_N_2_O_2_-H]^+^  [C_9_H_17_N_2_O_3_]^+^ | 183.1117  201.1235 | 2 | 13192 | PubChem | Oligopeptide |
| 231 | 7.8 | Vanilloylglycine | C_10_H_11_NO_5_ | 226.070999 | 226.0715 | 2.22 | [C_6_H_4_O_2_]^+^  [C_8_H_7_O_3_]^+^ | 108.0207  151.0396 | 2 | 4792 | HMDB | Metabolite of dietary polyphenols |
| 238 | 8.0 | Pro-Gly-Ile-Ile | C_19_H_34_N_4_O_5_ | 399.260197 | 399.2600 | 0.49 | [C_7_H_11_N_2_O_2_+H]+H^+^  [C_12_H_21_N_2_O_2_-2H]^+^  [C_13_H_22_N_3_O_3_]^+^  [C_13_H_23_N_3_O_4_]+H^+^ | 157.0975  223.1435  268.1645  286.1741 | 2 | 4959 | NIH | Oligopeptide |
| 239 | 8.0 | Castanospermine | C_8_H_15_NO_4_ | 190.107384 | 190.1077 | 1.66 | [C_5_H_9_O_3_+2H]+H^+^ | 120.0779 | 2 | 5593 | KEGG | Anti-inflammation, antiviral |
| 240 | 8.1 | Glu-Val-Phe | C_19_H_27_N_3_O_6_ | 394.197262 | 394.1974 | 0.35 | [C_8_H_9_N]+H^+^  [C_10_H_12_N_2_O-H]^+^  [C_9_H_14_N_2_O_3_+2H]+H^+^  [C_12_H_14_N_2_O_2_-H]^+^ | 120.0811  175.0874  201.1229  271.0959 | 2 | 11312 | HMDB |  |
| 241 | 8.1 | Phe-Thr-Ala-Trp | C_27_H_33_N_5_O_6_ | 524.250360 | 524.2491 | -2.4 | [C_8_H_10_N]^+^  [C_10_H_12_N_2_O]+H^+^  [C_16_H_22_N_3_O_4_]^+^ | 120.0803  177.1027  320.1583 | 2 | 5500 | PubChem | Oligopeptide |
| 244 | 8.3 | (Z)-4-[4-[[(E)-3-carboxyprop-2-enoyl]amino]anilino]-4-oxobut-2-enoic acid | C_14_H_12_N_2_O_6_ | 305.076813 | 305.0774 | 1.9 | [C_9_H_6_NO]^+^  [C_10_H_6_NO_2_]^+^  [C_10_H_7_N_2_O_2_+H]+H^+^  [C_10_H_7_NO_3_]+H^+^  [C_12_H_10_N_2_O_2_]+H^+^ | 144.0450  172.0389  189.0662  190.0449  215.0817 | 2 | 7339 | PubChem | - |
| 245 | 8.3 | N-formyl-N-hydroxy-3-cyclopentylmethyl-beta-alanine | C_10_H_17_NO_4_ | 216.123034 | 216.1229 | -0.62 | [C_9_H_15_NO-H]^+^  [C_9_H_15_NO]+H^+^ [C_9_H_16_NO_2_]^+^  [C_10_H_16_NO_3_]^+^ | 152.1057  154.1237  170.1158  198.1109 | 2 | 5610 | HMDB | Peptide derivatives |
| 247 | 8.4 | Palmitoleoylglycine | C_18_H_33_NO_3_ | 312.253320 | 312.2520 | -4.23 | [C_8_H_13_NO_2_+2H]+H^+^  [C_9_H_15_NO_2_+2H]+H^+^ | 158.1163  172.1328 | 2 | 5552 | HMDB | Antioxidant, anti-inflammatory, cryoprotective, and immunomodulatory |
| 252 | 8.7 | 2-[2-[tert-butoxycarbonyl(methyl)amino]propanoyl-methyl-amino]-3-methyl-4-methylsulfonyl-butanoic acid | C_16_H_30_N_2_O_7_S | 395.184648 | 395.1860 | 3.4 | [C_5_H_7_NO_3_]+H^+^  [C_7_H_12_N_2_O_3_-H]^+^  [C_8_H_12_N_2_O_4_-H]^+^  [C_8_H_12_N_2_O_5_]+H^+^  [C_9_H_17_NO_3_S-H]^+^ | 130.0500  171.0763  199.0699  217.0810  218.0846 | 2 | 6127 | PubChem | - |
| 256 | 8.9 | Pyroglutamylphenylalanine | C_14_H_16_N_2_O_4_ | 277.118283 | 277.118 | -1.02 | - | - | 3 | 4622 | HMDB | Peptide derivatives |
| 264 | 9.4 | L-lysyl-L-lysyl-glycyl-L-glutamine | C_19_H_37_N_7_O_6_ | 460.287808 | 460.2884 | 1.29 | - | - | 3 | 2162 | NIH | Oligopeptide |
| 266 | 9.5 | Lumichrome | C_12_H_10_N_4_O_2_ | 243.087652 | 243.0873 | -1.45 | [C_10_H_9_N_3_]+H^+^  [C_10_H_10_N_3_O+H]+H^+^  [C_11_H_9_N_3_O]+H^+^  [C_11_H_10_N_3_O_2_]^+^  [C_11_H_10_N_3_O_2_]+H^+^  [C_11_H_10_N_3_O_2_+H]+H^+^ | 172.0870  190.0977  200.0829  216.0759  217.0863  218.0920 | 2 | 3671 | HMDB | Yellow-green fluorescent compound  (Responsible for the coloration of the extract) |
| 269 | 9.6 | Nonate | C_9_H_16_O_4_ | 189.112135 | 189.1123 | 0.87 | [C_8_H_14_O-H]^+^ | 125.0961 | 2 | 11068 | HMDB | Energy storage |
| 278 | 10.0 | Peperinic acid | C_10_H_14_O_3_ | 183.101571 | 183.1017 | 0.7 | [C_9_H_13_-2H]^+^  [C_9_H_14_O-H]^+^  [C_10_H_13_O-2H]^+^ | 119.0849  137.0964  147.0792 | 2 | 4498 | HMDB | Nutrient |
| 280 | 10.2 | Decanedioic acid | C_10_H_18_O_4_ | 203.127786 | 203.1267 | -5.3 | [C_9_H_18_O-3H]^+^  [C_10_H_18_O_3_+H]^+^ | 139.1091  187.0206 | 2 | 4482 | MoNA | Degradation product of longer-chain fatty acids |
| 292 | 10.7 | 17-Hydroxylinolenic acid | C_18_H_30_O_3_ | 295.226771 | 295.2261 | -2.27 | [C_8_H_13_O]^+^  [C_10_H_14_-H]^+^  [C_11_H_16_-H]^+^  [C_10_H_15_O]^+^  [C_11_H_17_O]^+^  [C_18_H_29_O_2_]^+^ | 125.0976  133.1005  147.1155  151.1111  165.1271  277.2143 | 2 | 29974 | HMDB | Cardio-protection |
| 296 | 10.7 | ​(9Z,12E)-15,16-dihydroxyoctadeca-9,12-dienoic acid | C_18_H_32_O_4_ | 313.237336 | 313.2313 | -19.3 | [C_9_H_16_O-H]^+^  [C_18_H_32_O_4_+H]^+^ | 139.1072  313.1660 | 2 | 3671 | MoNA | Oxidized metabolite of linoleic acid |
| 302 | 11.0 | 2-[1-[4-[2-(2-Methylprop-2-enoyloxy)pentan-2-yl]phenoxy]ethoxy]ethyl adamantane-1-carboxylate | C_30_H_42_O_6_ | 499.305416 | 499.3043 | -2.2 | [C_8_H_12_O]+H^+^  [C_8_H_12_O_2_-H]^+^  [C_13_H_18_O-H]^+^  [C_14_H_16_O_2_]+H^+^  [C_15_H_19_O_2_]^+^  [C_14_H_18_O_3_]+H^+^  [C_15_H_19_O_3_+H]+H^+^  [C_23_H_30_O_5_]+H^+^ | 125.0962  139.0751  189.1256  217.1216  231.1366  235.1323  249.1471  387.2162 | 2 | 10350 | PubChem | - |
| 305 | 11.1 | 2-amino-3-hydroxybutanoic acid;2-amino-3-methylbutanoic acid | C_9_H_20_N_2_O_5_ | 237.144498 | 237.1446 | 0.46 | - | - | 3 | 2306 | NIH | - |
| 308 | 11.1 | Phe-His-Ile-Pro | C_26_H_36_N_6_O_5_ | 513.281995 | 513.2809 | -2.1 | [C_6_H_7_N_3_O+H]+H^+^  [C_17_H_26_N_5_O_4_]+H^+^ | 139.0750  365.2055 | 2 | 5227 | NIH | Oligopeptide |
| 309 | 11.1 | Anabsinthin | C_30_H_40_O_6_ | 497.289765 | 497.2888 | -1.94 | [C_8_H_12_O]+H^+^  [C_8_H_9_O_2_+H]+H^+^  [C_23_H_27_O_5_+H]+H^+^ [C_25_H_31_O_6_]^+^  [C_30_H_39_O_5_]^+^ | 125.0948  139.0763  385.2011  427.2142  479.2793 | 2 | 7364 | HMDB | Anti-inflammatory and bitter tasting agent |
| 322 | 11.5 | Thr-Lys-Pro-Arg | C_21_H_40_N_8_O_6_ | 501.314358 | 501.3166 | 4.5 | - | - | 3 | 1638 | HMDB | Peptide related to immune function |
| 323 | 11.6 | Cyclo(Gly-Leu-Gly-:Leu-Gly) | C_18_H_31_N_5_O_5_ | 398.239796 | 398.2414 | 4.03 | - | - | 3 | 3819 | HMDB | Oligopeptide |
| 329 | 11.9 | Trp-Ala-Arg | C_20_H_29_N_7_O_4_ | 432.235379 | 432.2356 | 0.51 | - | - | 3 | 1680 | NIH | Oligopeptide |
| 332 | 11.9 | Arg-Val-Ile | C_17_H_34_N_6_O_4_ | 387.271430 | 387.2726 | 3.02 | - | - | 3 | 1506 | NIH | Oligopeptide |
| 339 | 12.2 | L-Ascorbic acid, 6-octadecanoate | C_24_H_42_O_7_ | 443.300330 | 443.2996 | -1.65 | [C_6_H_9_O_3_-2H]^+^  [C_15_H_25_O_5_]^+^ | 127.0386  285.1691 | 2 | 7933 | KEGG | Vitamin C, antioxidant |
| 342 | 12.2 | Acetal R | C_13_H_20_O_2_ | 209.153606 | 209.1531 | -2.42 | [C_9_H_10_O_2_]+H^+^  [C_9_H_10_O_2_+2H]+H^+^  [C_10_H_13_O_2_+H]+H^+^  [C_11_H_15_O_2_]^+^  [C_12_H_17_O_2_]^+^  [C_12_H_17_O_2_]+H^+^ | 151.0760  153.0905  167.1055  179.1053  193.1216  194.1283 | 2 | 14872 | HMDB | Green, herbal, and hyacinth tasting agent |
| 355 | 12.7 | FA 18:3+1O | C_18_H_30_O_3_ | 277.218290 | 277.2145 | -13.7 | - | - | 3 | 4325 | MoNA | Oxidized derivative of a polyunsaturated fatty acid |
| 360 | 12.8 | (9Z,11E,13S)-13-hydroperoxyoctadeca-9,11-dienoic acid | C_18_H_32_O_4_ | 313.237336 | 313.2352 | -6.82 | [C_9_H_14_-H]^+^  [C_10_H_16_-H]^+^  [C_9_H_15_O]^+^  [C_11_H_18_O-H]^+^  [C_18_H_31_O_3_]^+^ | 121.1104  135.1154  139.1104  165.1269  295.2258 | 2 | 5352 | HMDB | Derivatives of linoleic acid |
| 363 | 12.8 | Armillane | C_23_H_32_O_7_ | 421.222080 | 421.2235 | 3.37 | - | - | 3 | 1197 | HMDB | - |
| 368 | 13.0 | Leu-Asn-His | C_16_H_26_N_6_O_5_ | 383.203744 | 383.2024 | -3.5 | [C_11_H_16_N_6_O_3_]+H^+^  [C_14_H_20_N_4_O_4_]^+^  [C_12_H_17_N_6_O_5_+H]+H^+^ | 281.1344  308.1457  327.1392 | 2 | 6564 | PubChem | Oligopeptide |
| 371 | 13.1 | Aspergillusenes A | C_15_H_22_O_2_ | 235.169256 | 235.1681 | -4.92 | [C_7_H_10_O_2_-3H]^+^  [C_11_H_18_O_2_-3H]^+^ | 123.0434  179.1064 | 2 | 9645 | HMDB | Antibacterial, antifungal, anticancer, anti-inflammatory, antioxidant |
| 372 | 13.1 | 5,7,3',4'-Tetrahydroxyflavanone 7-alpha-L-arabinofuranosyl-(1->6)-glucoside | C_24_H_28_N_2_O | 361.227440 | 361.2282 | 2.1 | - | - | 3 | 2578 | HMDB | - |
| 379 | 13.5 | 1,2,3-Tris(5-methoxypentyl)guanidine | C_19_H_41_N_3_O_3_ | 360.322069 | 360.3225 | 1.2 | - | - | 3 | 3400 | NIH | - |
| 385 | 13.7 | Linolenic acid | C_18_H_30_O_2_ | 279.231857 | 279.2319 | 0.15 | [C_8_H_13_]^+^  [C_9_H_12_]+H^+^  [C_10_H_15_]^+^ | 109.1004  121.1027  135.1157 | 2 | 10239 | HMDB | Cardio-protection |
| 387 | 13.8 | His-Thr-Ala-Pro | C_18_H_28_N_6_O_6_ | 425.214309 | 425.2127 | -3.78 | [C_11_H_15_N_6_O_3_+H]+H^+^  [C_12_H_17_N_6_O_4_]^+^  [C_16_H_24_N_6_O_4_]+H^+^  [C_14_H_19_N_6_O_6_+H]+H^+^ | 281.1340  309.1282  365.1919  369.1525 | 2 | 31845 | NIH | Oligopeptide |
